# Movement organization and neuromuscular coordination underlying offensive performance in para-fencing

**DOI:** 10.3389/fspor.2026.1802474

**Published:** 2026-04-15

**Authors:** Nawfal Mahdi, Udai Mahdi, Inaam J. Sadiq, Rafid Qaduri, Noora Mustafa, Maher Asi, Mohammed Bader, Safaa A. Ismaeel

**Affiliations:** 1 College of Physical Education and Sports Sciences, University of Diyala, Diyala, Iraq; 2 College of Engineering, Mustansiriah University, Baghdad, Iraq; 3 Directorate of Education of Diyala, Diyala, Iraq; 4 College of Physical Education and Sports Sciences, Mustansiriah University, Baghdad, Iraq

**Keywords:** inertial measurement units, kinematic profiling, movement strategies, neuromuscular coordination, para-fencing, surface electromyography, trunk stability

## Abstract

**Background/objectives:**

Para-fencing performance emerges from the interaction of kinematic execution, neuromuscular coordination, and mechanical constraints rather than from execution speed alone. Understanding how these factors combine into distinct performance strategies is essential for individualized assessment and training in adaptive sports. The primary objective of this study was to identify distinct performance strategies in para-fencers by deriving kinematic profiles from inertial measurement unit (IMU) data collected during a standardized offensive task. A secondary objective was to examine whether these strategies could be differentiated based on neuromuscular coordination patterns assessed using surface electromyography (sEMG), along with passive muscle mechanical properties and isometric strength measures.

**Methods:**

Thirty para-fencers performed repeated offensive actions toward a fixed target under controlled laboratory conditions. Upper-limb kinematics and trunk stability were quantified using IMUs, while sEMG was used to assess muscle activation timing, burst characteristics, and agonist–antagonist co-contraction. Passive muscle mechanical properties were evaluated using myotonometry, and isometric strength was measured with a calibrated force device. IMU-derived kinematic features were standardized and analyzed using cluster analysis to identify distinct performance strategies. Between-cluster comparisons were conducted for performance outcomes, neuromuscular variables, muscle mechanical properties, and strength measures.

**Results:**

Three distinct performance strategies were identified. One strategy was characterized by high peak angular velocity, increased trunk oscillation, and greater movement variability, reflecting a fast but variable execution pattern. A second strategy demonstrated smoother movement execution, reduced trunk sway, lower co-contraction levels, and superior accuracy, representing a stable–accurate profile. The third strategy exhibited intermediate characteristics, indicating a balance between execution speed and control. Neuromuscular coordination patterns, passive muscle mechanical properties, and isometric strength measures further differentiated the identified strategies.

**Conclusions:**

Para-fencing performance is organized through distinct movement strategies that reflect differences in kinematic control, neuromuscular coordination, and mechanical constraints rather than speed alone. In the context of this study, movement strategies refer to distinct patterns of movement organization that emerge from the interaction between kinematic execution, neuromuscular coordination, and underlying mechanical properties. These strategies reflect how athletes adapt their motor behavior to task-specific and environmental constraints in order to achieve performance goals. Profiling-based biomechanical approaches provide valuable insight into performance organization and support individualized assessment and training strategies in adaptive sports.

## 1 Introduction

In para-fencing, offensive performance extends beyond the simple expression of weapon speed or striking force (1). Within the specific constraints of wheelchair-based competition, movement execution depends on how upper-limb actions are coordinated with trunk control to ensure both speed and postural stability. The lack of lower-limb contribution places greater mechanical and control demands on the upper body, particularly during rapid attacking actions (2). As a result, relatively small variations in movement co-ordination or muscle mechanical behavior may translate into noticeable differences in performance effectiveness (3).

In para-fencing, athletes are usually placed into categories such as A, B, and C, mainly depending on trunk control and balance. In general, those in Category A tend to have better trunk stability and can reach more comfortably during actions, while athletes in the lower categories may face more limitations in maintaining posture (4).

At the same time, these categories are not only used for classification purposes. When observing performance more closely, the differences start to appear in how movements are organized and adjusted. With the limited role of the lower limbs, the trunk becomes more involved than one might expect. Athletes often rely on it during offensive actions, sometimes as a way to support or compensate for movement. This seems to have an effect on coordination, and possibly on how different movement strategies develop during the task.

Biomechanical research in fencing and related sports has often relied on group-averaged measures to describe movement characteristics (4–7). While such approaches provide general insights, they may obscure meaningful inter-individual differences in movement organization and coordination patterns, particularly in complex motor tasks. Although such analyses provide useful general descriptions, they may overlook meaningful inter-individual differences in how movements are organized. In practice, athletes performing the same task at comparable speeds often differ in accuracy, trunk control, and movement consistency across repetitions (8). These observations suggest that performance may be structured around distinct movement strategies rather than a single optimal execution pattern, a distinction that is not readily captured through averaged kinematic variables (9). In the context of this study, movement strategies refer to distinct patterns of movement organization that emerge from the interaction between kinematic execution, neuromuscular coordination, and underlying mechanical properties. These strategies reflect how athletes adapt their motor behavior to task-specific and environmental constraints in order to achieve performance goals.

Previous biomechanical studies in fencing have primarily focused on the analysis of lunge mechanics, upper-limb kinematics, and temporal characteristics of offensive actions (10). These studies have highlighted the importance of coordination between the upper limbs and trunk in achieving effective performance. In para-fencing, where lower-limb contribution is absent, the role of trunk stability and upper-limb coordination becomes even more critical (11). Recent advancements in wearable sensor technology, particularly inertial measurement units and surface electromyography, have enabled more detailed investigation of movement patterns and neuromuscular coordination in sport performance (4, 12). However, existing studies have typically examined these variables in isolation rather than integrating them within a unified analytical framework.

Recent applications of wearable inertial measurement units have allowed movement characteristics to be examined in greater detail (5). Variables such as angular velocity, temporal features, trunk oscillation, movement smoothness, and trial-to-trial variability offer insight into how movements are executed rather than merely how fast or how far they occur. When considered collectively, these features enable the construction of kinematic profiles that reflect strategy-dependent differences in movement organization. However, kinematic descriptions alone remain incomplete unless they are interpreted alongside the neuromuscular processes that shape movement execution (13).

From a neuromuscular perspective, fencing performance is strongly influenced by the timing and sequencing of muscle activation, as well as the degree of agonist–antagonist co-contraction required to stabilize joints during rapid actions. Variations in these coordination patterns may help explain why some athletes maintain accuracy and stability at higher execution speeds, whereas others exhibit increased movement variability or reduced control (8). Passive muscle mechanical properties and isometric force capacity further define the mechanical conditions under which neuromuscular control operates and may contribute to the selection of specific movement strategies (9).

Considering para-fencers in terms of distinct performance strategies represents a shift from describing average performance characteristics toward examining how athletes organize movement under task-specific constraints (4). A profiling-based approach offers the opportunity to identify patterns of execution that remain concealed in traditional analyses and to relate these patterns to underlying coordination mechanisms. Such information is relevant for both performance assessment and the design of individualized training interventions (14).

Accordingly, the purpose of this study was to derive kinematic profiles from inertial sensor data collected during a standardized offensive task in para-fencing and to classify athletes into distinct performance strategies (15). A secondary aim was to examine whether these strategies could be distinguished based on neuromuscular coordination patterns assessed using surface electromyography, together with measures of muscle mechanical properties and isometric strength. By combining kinematic, neuromuscular, and mechanical perspectives, this study seeks to provide a biomechanically grounded description of performance strategies in para-fencing (16). Despite the growing body of research examining kinematic and neuromuscular aspects of fencing and related sports, most studies have focused on isolated variables or group-averaged outcomes. Such approaches may overlook how athletes organize movement through distinct coordination patterns under task-specific constraints. In particular, there is a lack of integrative profiling approaches that combine kinematic data, neuromuscular coordination, and muscle mechanical properties to identify distinct performance strategies in para-fencing. This gap is especially relevant in wheelchair-based fencing, where movement organization is shaped by unique biomechanical constraints. Therefore, the present study aims to address this limitation by adopting a multidimensional profiling approach to classify performance strategies and examine their underlying neuromuscular and mechanical characteristics. Para-fencing is performed in a wheelchair-based setting in which athletes are fixed to the ground, and movement is primarily generated through the upper limbs and trunk. Athletes are classified according to functional ability, typically into categories based on the level of trunk control and balance. These classification differences are important because they influence movement strategies, neuromuscular coordination, and the ability to control body position during offensive actions. As a result, performance in para-fencing is strongly shaped by the interaction between mechanical constraints imposed by wheelchair fixation and the athlete's functional capabilities.

### 1.1 Purpose of the study

The primary purpose of this study was to identify distinct performance strategies in para-fencers by deriving kinematic profiles from inertial measurement unit data collected during a standardized offensive task. Specifically, the study aimed to classify athletes based on movement features reflecting execution speed, smoothness, trunk stability, and movement variability. A secondary purpose was to determine whether the identified performance strategies could be differentiated by neuromuscular coordination patterns assessed using surface electromyography, with additional consideration of passive muscle mechanical properties and isometric strength measures. Through this approach, the study sought to provide a biomechanical characterization of performance strategies that extends beyond group-averaged descriptions of movement.

### 1.2 Hypotheses

1. It was hypothesized that kinematic features derived from inertial measurement units would yield two or more distinct kinematic profiles representing different performance strategies during the offensive fencing task.
2. It was hypothesized that the identified performance strategies would differ in key kinematic characteristics, such that strategies characterized by lower movement variability, reduced trunk oscillation, and smoother movement patterns would demonstrate superior task performance compared with strategies exhibiting higher variability and less stable execution.
3. It was hypothesized that neuromuscular coordination patterns, including muscle activation timing, sequencing, and agonist–antagonist co-contraction, would differ between performance strategies and contribute to their differentiation.
4. It was hypothesized that passive muscle mechanical properties and isometric strength measures would further distinguish performance strategies, reflecting mechanical constraints that influence movement organization and execution.

## 2 Materials and methods

### 2.1 Participants

Thirty para-fencers (*n* = 30) volunteered to participate in this study. All participants were recruited from the International Center for the Care of Gifted Athletes with Disabilities, where they were actively engaged in structured fencing training programs (10). Participants had a minimum of two years of fencing experience and were free from acute musculoskeletal injuries at the time of testing as shown in Table 1. Prior to participation, all athletes were informed of the study procedures and provided written informed consent in accordance with institutional ethical standards. The study protocol was approved by the Institutional Review Board (IRB) of our institution (Protocol No. 2019P001025). All participants provided written informed consent, and the study was conducted in accordance with internationally accepted ethical standards for human research.

**Table 1:** Participant characteristics (mean ± SD).

Variable	Mean ± SD
Age (years)	24.6 ± 3.8
Body mass (kg)	71.2 ± 8.5
Height (cm)	173.4 ± 6.9
Training experience (years)	5.1 ± 1.9

Additional participant characteristics were recorded to improve the interpretability of the results. All participants were classified as Category A para-fencers, indicating a relatively higher level of trunk control and functional ability according to official classification standards. The sample consisted exclusively of male athletes, all of whom were right-hand dominant. All athletes performed the task with the foil weapon. Their consistent participation in organized training and competitive settings at a specialized center suggests an advanced level of performance. These features were considered relevant, as they may influence movement behavior, coordination patterns, and overall task execution. These characteristics were considered relevant due to their potential influence on movement organization, neuromuscular coordination, and task execution.

### 2.2 Instrumentation

#### 2.2.1 Inertial measurement units (IMU)

Kinematic data were collected using inertial measurement units capable of recording triaxial acceleration and angular velocity (12). IMUs were securely affixed to the trunk (sternum level), the upper arm of the weapon side, and the forearm of the weapon side using elastic straps to minimize sensor displacement during movement. Sensor placement was selected to capture upper-limb motion characteristics and trunk stability relevant to offensive fencing actions (17).

#### 2.2.2 Surface electromyography (sEMG)

Neuromuscular activity was recorded using a surface electromyography system. Bipolar electrodes were placed on the dominant-side muscles primarily involved in fencing actions, including the anterior deltoid, triceps brachii and erector spinae (18). Electrode placement followed standard anatomical guidelines, and skin preparation procedures were applied to reduce impedance and ensure signal quality.

Kinematic data were collected using an inertial measurement unit (IMU) system (SMART-DX, BTS Bioengineering Corp., Italy), which is commonly applied in sport biomechanics for detailed motion analysis. The system was used to capture segmental movement patterns during the execution of the fencing task.

Surface electromyographic (sEMG) signals were recorded using a wireless system from Noraxon (Noraxon USA Inc., USA), with MR3 software platform. This combination of systems made it possible to examine both movement characteristics and muscle activation within the same experimental setting. Sensor placement was based on the main body segments involved in the task. IMUs were positioned to capture trunk and upper-limb movement, while sEMG electrodes were placed over muscles directly contributing to force production and stabilization during the action.

#### 2.2.3 Muscle mechanical properties

Passive muscle mechanical properties were assessed using a handheld myotonometry device. Measurements were obtained from the anterior deltoid, triceps brachii, and erector spinae muscles of the dominant side. Variables extracted included muscle tone, stiffness, and decrement (19). Three consecutive measurements were recorded at each site, and the mean value was used for analysis.

#### 2.2.4 Isometric strength assessment

Isometric strength was measured using a calibrated force measurement device (20). Participants performed maximal voluntary isometric contractions for elbow extension and shoulder push or rotation actions relevant to fencing performance. Each test consisted of two to three maximal trials with adequate rest between attempts, and the highest recorded value was retained for analysis (21).

### 2.3 Procedures

**Figure 1:**
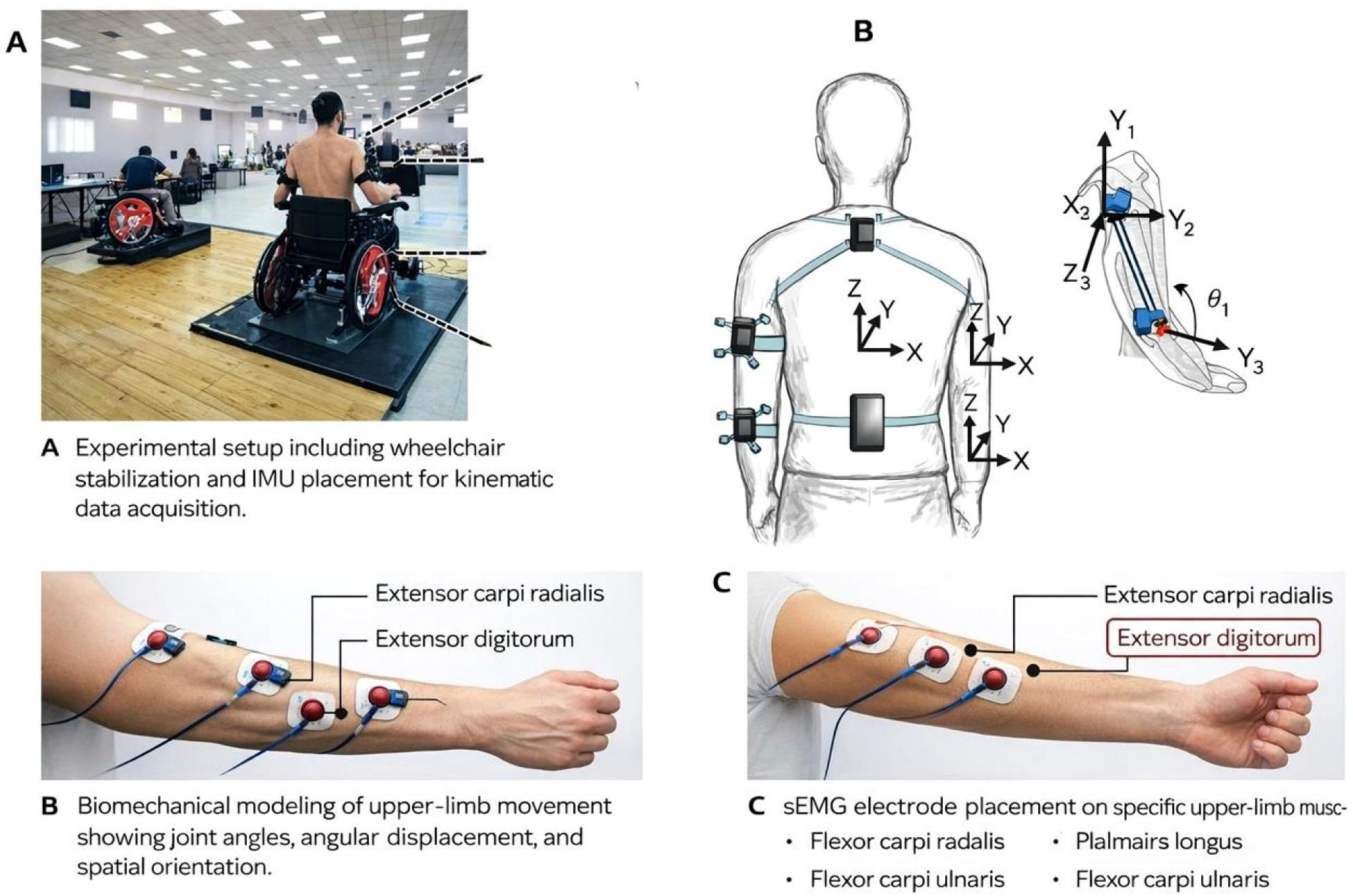
Experimental setup and sensor placement during the standardized offensive task in para-fencing. (**A**) Experimental setup showing wheelchair stabilization and placement of inertial measurement units (IMUs) on the upper arm, forearm, and trunk segments to capture kinematic data. (**B**) Biomechanical representation of upper-limb movement illustrating segmental orientation, joint angles, and spatial coordinate systems derived from IMU data. (**C**) Surface electromyography (sEMG) electrode placement on selected upper-limb muscles, including flexor and extensor muscle groups, for recording neuromuscular activity during task execution.

All testing was conducted during a single laboratory session lasting approximately 75–90 min. Participants first completed a standardized warm-up consisting of light upper-limb mobility exercises and submaximal fencing movements. Participants performed the task under standardized conditions (see Figure 1). Following the warm-up, baseline assessments of muscle mechanical properties and isometric strength were performed. Subsequently, IMU and sEMG sensors were attached, and signal quality was verified.

Participants then performed a standardized offensive fencing task involving repeated direct attacks toward a fixed dummy target positioned at a distance of 1.5 m from the athlete, corresponding to a typical reach distance in para-fencing practice. All participants executed the task using the foil weapon. The wheelchair was securely fixed to the ground using a rigid frame system to prevent displacement during movement execution. The task was carried out under three speed conditions: controlled (accuracy-focused), preferred, and maximal speed. Each condition consisted of eight to ten trials, with short rest intervals between trials and longer rest periods between speed conditions to minimize fatigue effects (22). Task performance was quantified using accuracy scores and temporal measures derived from kinematic data, and all trials were visually monitored to ensure consistency of execution (19).

### 2.4 Signal processing

#### 2.4.1 IMU data

Raw IMU signals were filtered using a low-pass Butterworth filter. Kinematic variables were derived from angular velocity and acceleration data, including peak angular velocity, time-to-peak velocity, time-to-target, trunk oscillation amplitude, movement smoothness (jerk index), and trial-to-trial variability. All variables were normalized where appropriate to allow inter-individual comparison (12).

#### 2.4.2 sEMG data

sEMG signals were band-pass filtered, rectified, and smoothed using a root mean square (RMS) algorithm. Muscle activation onset times were identified using a threshold-based method relative to baseline activity. Additional variables included peak RMS amplitude, burst duration, and agonist–antagonist co-contraction indices calculated between the biceps brachii and triceps brachii (2).

### 2.5 Statistical analysis

Basic descriptive statistics were first computed for all variables, with results expressed as mean ± standard deviation. To check whether the data met the assumptions for parametric testing, normality was examined using the Shapiro–Wilk test. Prior to further analysis, the kinematic variables obtained from the IMUs were standardized using z-scores to allow for meaningful comparisons across participants. A clustering procedure was then applied in order to explore potential groupings of movement behavior. The number of clusters retained was guided by both their interpretability and commonly used internal validity criteria. Differences between the identified groups were examined using one-way ANOVA when the data were normally distributed, whereas the Kruskal–Wallis test was used in cases where these assumptions were not satisfied. When statistically significant differences were observed, appropriate *post hoc* comparisons were carried out. In addition to *p*-values, Effect sizes were calculated and reported (*η*^2^ for ANOVA and r for non-parametric tests) to provide additional insight into the magnitude of the observed differences. The level of statistical significance was set at *p* < 0.05.

Given the exploratory nature of the study, the sample size (*n* = 30) was considered adequate for identifying meaningful patterns within the dataset. Similar sample sizes have been reported in previous biomechanical studies employing sensor-based and clustering approaches. Moreover, the relatively consistent background of the participants, particularly in terms of training and functional classification, was expected to support the stability of the identified clusters.

## 3 Results

### 3.1 IMU-derived kinematic profiles and performance strategy clustering

Table 2 presents the IMU-derived kinematic features used to identify distinct performance strategies among the para-fencers. Cluster analysis of standardized kinematic variables revealed three distinct clusters reflecting different movement execution patterns.

**Table 2:** IMU-derived kinematic features used for clustering (mean ± SD).

Variable	Cluster A (Fast–Noisy)	Cluster B (Stable–Accurate)	Cluster C (Controlled–Efficient)
Peak angular velocity (deg/s)	812 ± 94	685 ± 76	724 ± 81
Time-to-peak velocity (ms)	148 ± 21	172 ± 25	160 ± 22
Jerk index (a.u.)	0.82 ± 0.14	0.46 ± 0.09	0.58 ± 0.11
Trunk sway amplitude (deg)	6.9 ± 1.2	3.8 ± 0.8	4.6 ± 0.9
Movement variability (CV %)	18.4 ± 3.1	9.6 ± 2.4	12.1 ± 2.7

**Figure 2:**
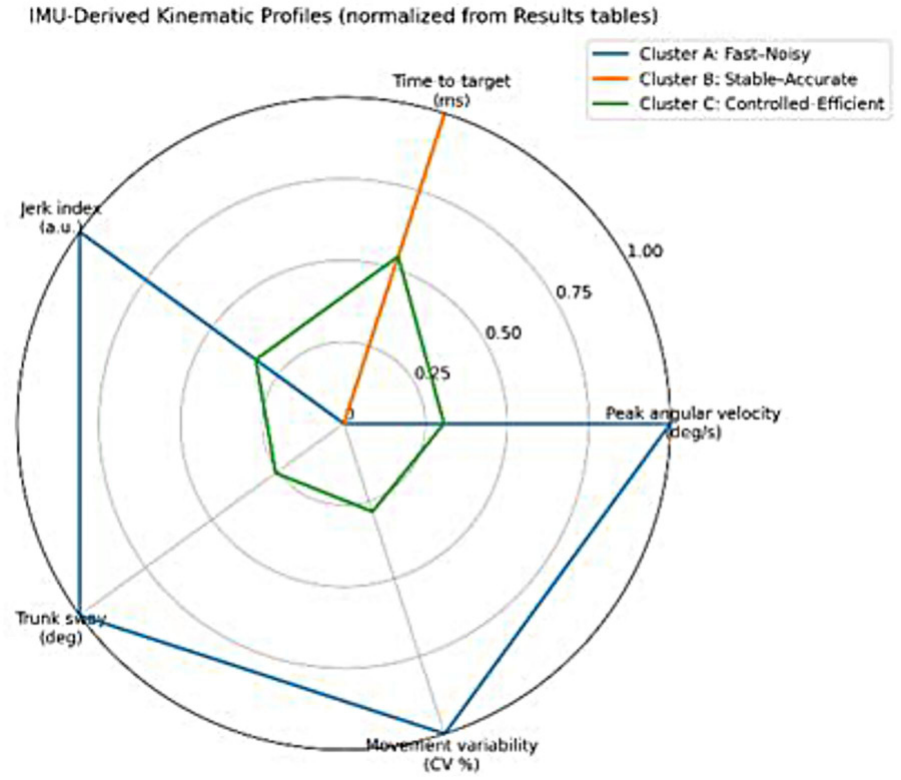
Radar plot illustrating IMU-derived kinematic profiles for the identified performance strategies in para-fencers. Each profile represents a distinct combination of execution speed, movement smoothness, trunk stability, and movement variability during the standardized offensive task.

The identified clusters differed primarily in movement smoothness, trunk stability, and trial-to-trial variability. Cluster A exhibited the highest execution speed but also the greatest movement variability and trunk sway. Cluster B demonstrated smoother movement patterns with enhanced trunk stability, whereas Cluster C showed intermediate characteristics, reflecting a controlled and efficient execution profile. Figure 2 illustrates the IMU-derived kinematic profiles corresponding to the identified performance strategies.

### 3.2 Task performance outcomes across identified strategies

Task performance measures across the identified performance strategies are summarized in Table 3.

**Table 3:** Task performance across identified performance strategies.

Variable	Cluster A	Cluster B	Cluster C	*p*-value
Accuracy score (0–10)	6.1 ± 1.2	8.7 ± 0.9	7.8 ± 1.0	<0.001
Time-to-target (ms)	410 ± 48	462 ± 55	438 ± 50	0.012

Cluster B demonstrated significantly higher accuracy scores compared with the other clusters, whereas Cluster A achieved shorter time-to-target values, indicating a speed-dominant execution strategy.

### 3.3 Neuromuscular coordination patterns

Table 4 summarizes the neuromuscular coordination variables derived from surface electromyography across the identified performance strategies.

**Table 4:** Neuromuscular coordination variables derived from surface electromyography across the identified performance strategies.

Variable	Cluster A	Cluster B	Cluster C	*p*-value
Deltoid onset (ms)	−42 ± 18	−61 ± 14	−54 ± 16	0.021
Triceps onset (ms)	−18 ± 15	−39 ± 13	−31 ± 14	0.017
Co-contraction index (%)	38.6 ± 6.9	24.3 ± 5.1	29.8 ± 5.7	<0.001
Burst duration (ms)	286 ± 34	242 ± 28	258 ± 31	0.032

Significant differences were observed between clusters in muscle activation timing and agonist–antagonist co-contraction patterns. Cluster B exhibited earlier muscle onsets and lower co-contraction levels, reflecting more efficient neuromuscular coordination.

### 3.4 Muscle mechanical properties and isometric strength

Passive muscle mechanical properties measured across the identified performance strategies are presented in Table 5.

**Table 5:** Muscle mechanical properties across strategies.

Variable	Cluster A	Cluster B	Cluster C	*p*-value
Deltoid stiffness (N/m)	392 ± 44	328 ± 36	354 ± 39	0.008
Triceps stiffness (N/m)	415 ± 51	346 ± 42	372 ± 46	0.011
Muscle tone (Hz)	16.9 ± 1.8	14.8 ± 1.5	15.6 ± 1.6	0.019

Isometric strength measures across strategies are reported in Table 6.

**Table 6:** Isometric strength measures across strategies.

Variable	Cluster A	Cluster B	Cluster C	*p*-value
Elbow extension MVC (N)	412 ± 52	366 ± 48	384 ± 50	0.041
Shoulder push MVC (N)	438 ± 61	392 ± 55	414 ± 58	0.048

Clusters differed significantly in both passive muscle mechanical properties and isometric strength. Cluster A exhibited higher stiffness and strength values, whereas Cluster B demonstrated lower stiffness and strength, consistent with its stable and accuracy-oriented performance profile.

## 4 Discussion

Performance analysis in para-fencing revealed that athletes did not adopt a single uniform movement pattern during offensive actions (23). Instead, the kinematic data derived from inertial sensors demonstrated clear strategy-dependent differences in how movements were executed. As shown in Table 2, variations were observed in execution speed, trunk stability, and movement variability, indicating that performance organization differed substantially between athletes even when performing the same task (7). A closer examination of the kinematic profiles suggests that higher movement speed alone did not guarantee effective task execution (11). Although participants classified within Cluster A achieved shorter time-to-target values, this was accompanied by greater trunk oscillation and increased trial-to-trial variability. In contrast, athletes in Cluster B displayed smoother movement trajectories and reduced variability, which coincided with higher accuracy scores (Table 3). Within this task context, accuracy outcomes were more closely aligned with stability-related kinematic features (6). Neuromuscular coordination patterns further differentiated the identified strategies. Differences in muscle activation timing and co-contraction levels (Table 4) indicate that athletes adopting more stable strategies relied on a more economical organization of muscle activity. Reduced agonist–antagonist co-contraction may reflect a refined control strategy that limits unnecessary joint stiffening and allows for smoother force transmission. Conversely, elevated co-contraction observed in faster strategies may represent a compensatory response to increased mechanical demands imposed by rapid movement execution (6). Mechanical properties of the muscles provided additional context for these findings. As reported in Table 5, athletes exhibiting higher passive muscle stiffness and tone tended to demonstrate less consistent movement patterns. Increased passive resistance may limit fine adjustments during fast offensive actions, thereby influencing overall movement quality (24). While greater isometric strength was observed in some faster strategies (Table 6), strength capacity alone was not sufficient to produce superior accuracy, emphasizing the role of coordination and mechanical compliance in performance regulation. Across the present sample, performance differences were consistently associated with variations in movement organization rather than execution speed alone. The identification of distinct performance strategies supports the use of profiling approaches to capture how athletes adapt movement organization to task-specific constraints. From a biomechanical standpoint, this perspective provides a more detailed understanding of performance behavior than traditional group-averaged analyses and offers a practical basis for individualized assessment and training (25). When these findings are viewed within the specific context of para-fencing, some of the observed patterns become more intuitive. Unlike able-bodied fencing, movement here is largely constrained by wheelchair fixation, which shifts the primary demand toward trunk control and upper-limb coordination. Under such conditions, even small variations in trunk stability can influence both accuracy and movement consistency. This may help explain why athletes in the stable–accurate group showed lower trunk oscillation and more efficient activation patterns, whereas those adopting faster execution appeared to rely on higher levels of co-contraction, possibly as a compensatory strategy to maintain joint stability during rapid actions. Similar observations have been reported in upper-body dominant and constrained movement settings, where coordination efficiency rather than speed alone plays a central role in performance outcomes (11, 12). Looking at these results in a more practical way, it doesn't really make sense to treat all athletes the same in training. Even when they perform the same task, there are clear differences in how they move, and those differences seem to matter. Some athletes rely more on speed but show less consistency, so they may need to spend more time improving trunk stability and the timing of muscle activation. Others already move in a more controlled way, and for them the focus might be on gradually increasing speed without losing that control.

This is where wearable systems start to become genuinely useful rather than just technically interesting. Tools such as IMUs and surface EMG do not simply generate data; they make it possible to track how an athlete's movement pattern shifts over time, sometimes in subtle ways that are not easily visible to the coach. That kind of feedback can support more informed adjustments in training, especially when dealing with athletes who operate under mechanical constraints like those seen in para-fencing. In fact, recent work in sports biomechanics has been moving in this direction less emphasis on one-size-fits-all programs, and more attention to how individual movement solutions emerge under specific constraints (4, 6).

### 4.1 Limitations and future directions

The findings of this study are specific to the experimental context in which the data were collected. All participants were drawn from the same training environment and were tested under identical preparation conditions. This facilitated controlled comparisons between athletes but also limited the range of training backgrounds and competitive experiences represented. It is therefore possible that different movement strategies would emerge in para-fencers exposed to alternative coaching systems or competition structures. The task selected for analysis was intentionally standardized to allow clear examination of movement execution. While this approach was effective for identifying strategy-related differences, it did not reproduce the adaptive demands of opponent-driven fencing actions. Some coordination behaviors that occur during reactive exchanges may not have been expressed under these conditions. In addition, measurements were confined to selected upper-limb and trunk muscles, providing focused insight into key contributors to performance rather than a complete representation of whole-body involvement. Future work would benefit from extending this approach to more diverse athlete groups and testing environments. Including reactive task constraints, multiple fencing actions, and repeated assessments over time may help clarify whether the identified performance strategies remain consistent or evolve with training and competitive exposure. Such extensions would strengthen the ecological relevance of kinematic and neuromuscular profiling in para-fencing.

## 5 Conclusions

This study demonstrated that para-fencing performance during a standardized offensive task is organized around distinct movement strategies rather than a single uniform execution pattern. By integrating IMU-derived kinematic profiling with neuromuscular coordination, passive muscle mechanical properties, and isometric strength measures, three clearly differentiated performance strategies were identified. These strategies reflected meaningful differences in movement smoothness, trunk stability, coordination efficiency, and mechanical constraints, extending beyond simple variations in execution speed.

Athletes exhibiting a stable–accurate strategy achieved superior task accuracy through smoother kinematic execution, reduced trunk sway, earlier muscle activation, and lower agonist–antagonist co-contraction. In contrast, faster strategies were associated with greater movement variability, increased trunk oscillation, and elevated co-contraction, suggesting compensatory control mechanisms under higher mechanical demands. Differences in passive muscle stiffness and strength further contributed to strategy differentiation, highlighting the interaction between neuromuscular control and underlying mechanical properties in shaping movement organization.

Overall, these findings emphasize that effective para-fencing performance depends on how movement is coordinated and stabilized rather than on speed alone. Profiling-based biomechanical approaches using wearable sensors offer a valuable framework for capturing inter-individual differences in performance organization and provide a practical basis for individualized assessment, targeted training, and adaptive performance optimization in para-fencing and other adaptive sports.

## Data Availability

The raw data supporting the conclusions of this article will be made available by the authors, without undue reservation.

## Supplementary material

Supplementary Table S1 Additional descriptive statistics of IMU-derived kinematic variables across performance strategies.
