# Supplementary material for: Movement organization and neuromuscular coordination underlying offensive performance in para-fencing
Source: Front Sports Act Living. 2026 Apr 15;8:1802474. doi: 10.3389/fspor.2026.1802474 (PMC13127253; doi:10.3389/fspor.2026.1802474)
Supplement: Supplementary Table S1 — Additional descriptive statistics of IMU-derived kinematic variables across performance strategies. [file table1.docx]

Table S1. Additional descriptive statistics of IMU-derived kinematic variables

| Variable | Statistic | Cluster A (Fast–Noisy) | Cluster B (Stable–Accurate) | Cluster C (Controlled–Efficient) |
| --- | --- | --- | --- | --- |
| Peak angular velocity (deg/s) | Mean ± SD | 812 ± 94 | 685 ± 76 | 724 ± 81 |
|  | Min–Max | 700–950 | 600–800 | 650–850 |
| Time-to-peak velocity (ms) | Mean ± SD | 148 ± 21 | 172 ± 25 | 160 ± 22 |
|  | Min–Max | 120–180 | 140–210 | 130–190 |
| Jerk index (a.u.) | Mean ± SD | 0.82 ± 0.14 | 0.46 ± 0.09 | 0.58 ± 0.11 |
|  | Min–Max | 0.60–1.00 | 0.30–0.60 | 0.40–0.75 |
| Trunk sway amplitude (deg) | Mean ± SD | 6.9 ± 1.2 | 3.8 ± 0.8 | 4.6 ± 0.9 |
|  | Min–Max | 5.0–8.5 | 2.5–5.0 | 3.5–6.0 |
| Movement variability (CV %) | Mean ± SD | 18.4 ± 3.1 | 9.6 ± 2.4 | 12.1 ± 2.7 |
|  | Min–Max | 14–24 | 6–14 | 9–17 |
